# Transcriptome Analysis of Immune Response of mIgM^+^ B Lymphocytes in Japanese Flounder (*Paralichthys olivaceus*) to *Lactococcus lactis in vitro* Revealed That IFN I-3 Could Enhance Their Phagocytosis

**DOI:** 10.3389/fimmu.2019.01622

**Published:** 2019-07-16

**Authors:** Xiaoqian Tang, Shun Yang, Xiuzhen Sheng, Jing Xing, Wenbin Zhan

**Affiliations:** ^1^Laboratory of Pathology and Immunology of Aquatic Animals, KLMME, Ocean University of China, Qingdao, China; ^2^Laboratory for Marine Fisheries Science and Food Production Processes, Qingdao National Laboratory for Marine Science and Technology, Qingdao, China

**Keywords:** phagocytosis, *Paralichthys olivaceus*, mIgM^+^ B lymphocytes, transcriptome, IFN I-3

## Abstract

B cells have recently been proven to have phagocytic activities, but few studies have explored the relevant regulation mechanisms. In this study, we showed that the Japanese flounder (*Paralichthys olivaceus*) membrane-bound (m)IgM^+^ B lymphocyte population could phagocytose inactivated *Lactococcus lactis* with a mean phagocytic rate of 25%. High-purity mIgM^+^ B lymphocytes were subsequently sorted to investigate the cellular response to *L. lactis* stimulation *in vitro*. Transcriptome analysis identified 1,375 differentially expressed genes (DEGs) after *L. lactis* stimulation, including 975 upregulated and 400 downregulated genes. Many of these DEGs were enriched in multiple pathways associated with phagocytosis such as focal adhesion, the phagosome, and actin cytoskeleton regulation. Moreover, many genes involved in phagolysosomal function and antigen presentation were also upregulated after stimulation, indicating that mIgM^+^ B lymphocytes may degrade the internalized bacteria and present processed antigenic peptides to other immune cells. Interestingly, the type I interferon 3 (*IFN I-3)* gene was upregulated after *L. lactis* stimulation, and further analysis showed that the recombinant (r)IFN I-3 significantly enhanced phagocytosis of *L. lactis* and *Edwardsiella tarda* by mIgM^+^ B lymphocytes. In addition, significantly higher intracellular reactive oxygen species (ROS) levels were detected in mIgM^+^ B lymphocytes following rIFN I-3 treatment. We also found that IFN I-3 significantly upregulated *Stat1* expression in mIgM^+^ B lymphocytes, and the enhancing effect of IFN I-3 on mIgM^+^ B lymphocyte-mediated phagocytosis was suppressed by fludarabine treatment. Collectively, these results demonstrate that mIgM^+^ B cell-mediated phagocytosis in the Japanese flounder is effectively triggered by bacterial stimulation, and further enhanced by IFN I-3, which itself may be regulated by Stat1.

## Introduction

The fish immune system has both adaptive and innate components. The innate immune system represents the first line of defense against pathogenic organisms and other foreign materials, and also plays an important role in driving adaptive immunity (1). Phagocytosis is one of the most important innate immune processes, playing a vital role in resisting pathogen invasion and triggering the adaptive immune response (2, 3). Phagocytosis is mainly accomplished by professional phagocytes such as macrophages, neutrophils, and dendritic cells (DCs) (4). Early studies on primary mammalian B cells revealed that unlike professional phagocytes, this immune subset lacks phagocytic activity and is instead the major initiator of humoral immunity (5). However, recent studies have revealed that fish B cells also possess potent phagocytic activity, similar to that of macrophages (6–10). These findings were subsequently extended to B cells originating from amphibians (e.g., *Xenopus laevis*) and reptiles (e.g., *Trachemys scripta*). However, the phagocytic abilities of B cells derived from these species are significantly lower than those of fish B cells (6, 11). Mouse B1 cells were found to have phagocytic activity (12, 13), while Raji B cells in humans actively phagocytose both live and dead *Mycobacterium tuberculosis* (14). Moreover, B cells can initiate the adaptive immune response by destroying the phagocytosed bacteria and presenting processed antigens to CD4^+^ T cells (12, 15). This evidence collectively demonstrates that vertebrate B cells possess phagocytic ability and play important roles in both innate and adaptive immunity.

Although current evidence suggests that phagocytic B cells are widely present in teleosts, their phagocytic activity varies among different species. Previous studies have shown that the phagocytosis rates of membrane-bound IgM-positive (mIgM^+^) B lymphocytes in Atlantic cod (*Gadus morhua*) and half-smooth tongue sole (*Cynoglossus semilaevis*) were lower than those in Atlantic salmon (*Salmo salar*) and lumpfish (*Cyclopterus lumpus*) (7–9). Moreover, the phagocytic rates exhibited by mIgM^+^ B lymphocytes in peripheral blood were higher than those in the pronephros (6, 10). Interestingly, B1 cells from obese mice exhibit stronger phagocytic activity, suggesting that a high-fat diet can affect phagocytosis in these cells (13). Moreover, B cells exposed to different phagocytic particles tend to exhibit different phagocytic activities. mIgM^+^ B lymphocytes from sea bass (*Lateolabrax japonicus*) phagocytosed *Lactococcus lactis* at a much lower rate than fluorescent microspheres (10). In contrast, mouse B1 cells displayed much higher phagocytic rates when confronted with *Escherichia coli* compared to fluorescent microspheres (16). However, the lack of information about the B cell response following phagocytic particle ingestion limits our understanding of the mechanisms involved in B cell phagocytosis.

This study analyzed the phagocytic activity of mIgM^+^ B lymphocytes from the Japanese flounder. Initially, mIgM^+^ B lymphocytes were sorted from peripheral blood using immunomagnetic bead separation technology. Transcriptomics was subsequently used to investigate the mIgM^+^ B cellular response upon *L. lactis* stimulation *in vitro*, followed by the identification and characterization of differentially expressed genes (DEGs) and their functions in B cell-mediated phagocytosis. We envisage that this research will broaden our understanding of the regulation mechanisms governing B cell phagocytosis and provide further insight into the role of B cells in teleost innate immunity.

## Materials and Methods

### Ethics Statement

This study was carried out in strict accordance with the ethical standards and the guidelines of “Regulations for the Administration of Affairs Concerning Experimental Animals” documented by the State Science and Technology Commission of Shandong Province. This study was also approved by the Committee of the Ethics on Animal Care and Experiments at the Ocean University of China. Fish were anesthetized with ethyl 3-amino-benzoate-methanesulfonic acid (MS222) before sacrificing and handling.

### Fish

Japanese flounder (750 ± 50 g) were purchased from a fish farm in Rizhao, Shandong Province, China. The fish were maintained in tanks containing aerated sand-filtered seawater at 20 ± 0.5°C for 1 week prior to the experiments.

### Phagocytosis Assay

The *L. lactis* strains were grown in M17 broth supplemented with 0.5% (w/v) glucose (GM17) at 30°C without agitation. The *L. lactis* were collected and washed using phosphate-buffered saline (PBS) by centrifugation at 5,000 g for 10 min at 4°C, and then inactivated with 36% (w/v) absolute formaldehyde for 10 min at 4°C. The inactivated *L. lactis* were labeled with fluorescein isothiocyanate (FITC) as previously described, then stored in the dark at −20°C (10).

For the phagocytosis assay, leukocytes were isolated from the peripheral blood of three individual Japanese flounder by discontinuous Percoll (Pharmacia, USA) gradient (1.020/1.070) centrifugation, according to the method described by Li et al. (17). The phagocytosis assay was conducted as previously described (10). Briefly, isolated leukocytes in L-15 medium were counted and diluted to 1 × 10^7^ cells/ml. Following the addition of FITC-labeled *L. lactis*, 500 μl of the leukocyte suspension was added into the wells of a 24-well culture plate at a cell to bacteria ratio of 1:20, and then incubated for 3 h at 22°C. The non-ingested *L. lactis* were then removed by placing the cell suspension on top of a 1.077 Percoll gradient and centrifugation at 680 *g* for 10 min at 4°C. The collected cells were subsequently stained with a monoclonal antibody (mAb) specific for Japanese flounder IgM, produced previously in our laboratory (17). After washing with PBS, the cells were labeled with Alexa Fluor® 647 goat anti-mouse IgG (Invitrogen, USA) and analyzed using an Accuri C6 flow cytometer (Becton Dickinson. USA). In parallel, the cell suspension was used to coat glass slides and fixed with 4% paraformaldehyde for 20 min at 22°C. The slides were then stained with 4′,6-diamidino-2-phenylindole (DAPI) and mounted with 90% glycerin, prior to observation under a fluorescence microscope (EVOS™ FL Auto 2, Invitrogen, USA).

### Cell Sorting

The mIgM^+^ B lymphocytes were sorted from peripheral blood using an immunomagnetic bead separation method, as previously described with some modifications (18). Briefly, leukocytes isolated from the peripheral blood of eight fish were pooled t into one sample and incubated with the Japanese flounder IgM-specific mAb at 22°C for 1.5 h. The cells were then washed three times using magnetic activated cell sorting (MACS) buffer (PBS containing 2 mM ethylenediaminetetraacetic acid [EDTA] and 0.5% bovine serum albumin [BSA]). The leukocyte pellets were resuspended in MACS buffer (80 μl per 10^7^ cells) and incubated with goat anti-mouse IgG magnetic beads (20 μl per 10^7^ cells, Miltenyi Biotec, Germany) for 15 min at 4°C. After another washing step, the leukocytes were resuspended in MACS buffer (500 μl per 10^8^ cells) and filtered through a 40-μm nylon mesh. A MACS LS column (Miltenyi Biotech) was installed in a MACS separator according to the manufacturer's instructions and balanced with 3 ml MACS buffer. The leukocyte suspension was then passed through the column, and magnetically unlabeled leukocytes were washed off using MACS buffer. The MACS LS column was subsequently removed from the MACS separator, and the magnetically labeled leukocytes were collected. To obtain a high-purity mIgM^+^ B lymphocyte fraction, the magnetically labeled leukocytes were sorted for a second time using a new balanced MACS LS column. The purity of sorted mIgM^+^ B lymphocytes was determined by flow cytometry, an indirect immunofluorescence assay (IIFA), and real-time polymerase chain reaction (RT-PCR). A trypan blue (0.4%) exclusion assay was performed to determine the viability of the sorted cells. A primary mAb specific for Japanese flounder IgM and a secondary Alexa Fluor® 647-conjugated goat anti-mouse IgG antibody (Invitrogen, USA) were used for flow cytometry and IIFA. The B cell (*IgM, IgD*, and *CD79b*) and T cell (*CD3*ε, *CD4-2*, and *CD8*β) marker genes were used to examine the purity of the sorted cells. All RT-PCR primers were designed according to the corresponding sequences and are listed in Table S1.

### RNA Isolation and Illumina Sequencing

Sorted mIgM^+^ B lymphocytes from eight fish were pooled together and incubated with *L. lactis* in 24-well culture plates for 3 h at 22°C at a cell to bacteria ratio of 1:20. The wells containing mIgM^+^ B lymphocytes without *L. lactis* represented negative controls. Following incubation, the mIgM^+^ B lymphocytes were collected, and non-ingested bacteria were removed using a 1.077 Percoll gradient as described in the phagocytosis assay section. The mIgM^+^ B lymphocytes collected from the treatment and control groups were used for RNA extraction.

Total RNA was extracted from the sorted mIgM^+^ B lymphocytes using the RNeasy Plus Mini Kit (Qiagen, Germany) following the manufacturer's instructions. RNA quality was analyzed using a Bioanalyzer 2100 (Agilent Technologies, USA). RNA from each mixed sample was divided into two parts: one for transcriptome sequencing, and the other part for quantitative RT-PCR (qRT-PCR) to verify the reliability of DEG data obtained from transcriptome sequencing.

cDNA libraries were generated using the TruSeq™ RNA Sample Preparation Kit (Illumina, USA) following the manufacturer's instructions for transcriptome sequencing. Briefly, mRNA was isolated from total RNA using the poly-T oligo-attached magnetic beads. The first cDNA strand was synthesized using random hexamer primers and the Superscript III Reverse Transcriptase (Invitrogen, USA). Second cDNA strand synthesis, end repair, and ligation of the adaptor steps were subsequently carried out. Finally, sequencing was performed by the Biomaker Company (China) using an Illumina Hiseq2000 platform (Illumina, USA).

### Gene Annotation and Differential Expression Analysis

The whole genome sequence of the Japanese flounder was obtained from the US National Center for Biotechnology Information (NCBI) database (https://www.ncbi.nlm.nih.gov/genome/?term=Paralichthys%20olivaceus). After sequencing, clean reads were obtained to trim the raw data by removing adaptor and low-quality sequences (Q < 20), ambiguous nucleotides, and short reads (<30 bp), using the CLC Genomics Workbench (CLC bio, Denmark). Clean reads from the treatment and control groups were next aligned to the Japanese flounder genome using the TopHat bioinformatics tool (19).

Based on the beta negative binomial distribution model, the Cuffquant and Cuffnorm components of Cufflinks software were used to quantify the expression level of transcripts and genes through the positional information of the reads mapped to the reference genome (19). Fragments Per Kilobase of transcript per Million fragments mapped (FPKM) was used as an indicator of transcript or gene expression levels (19).

Differential expression analysis of mIgM^+^ B lymphocytes was performed using the EBSeq package due to the lack biological replicates (19). For differential expression analysis, the Benjamini–Hochberg calibration method was used to correct the *p*-value obtained from the original hypothesis test. The false discovery rate (FDR) was used to exclude false-positive interference in the DEG analysis. Referring to previous studies, genes with a fold change ≥2 and FDR < 0.01 were set as the threshold for significant differential expression (20–22).

### Kyoto Encyclopedia of Genes and Genomes (KEGG) Enrichment Analysis of DEGs

The KEGG database is used to systematically relate gene function to genomic information. DEGs associated with mIgM^+^ B lymphocytes of the Japanese flounder were aligned against the KEGG database. Pathway enrichment analysis was then performed to identify enriched signal transduction or metabolic pathways.

### qRT-PCR

To validate the reliability of the transcriptome sequencing-derived DEG data, 10 representative DEGs were selected for qRT-PCR analysis, which was performed according to established protocols with some modifications (23). Briefly, total RNA was extracted from sorted mIgM^+^ B lymphocytes using the RNeasy Plus Mini Kit (Qiagen, Germany) following the manufacturer's instructions. RNA quality was assessed using a Bioanalyzer 2100 (Agilent Technologies, USA). qRT-PCR was carried out using SYBR GreenIMaster (Roche, Switzerland) in a LightCycler® 480 II Real Time System (Roche, Switzerland). Each assay was performed in triplicate with the *18s rRNA* gene as the internal reference. All data were analyzed relative to the *18s rRNA* gene using the 2^−ΔΔ*Ct*^ method. Differences between the treatment and control groups were investigated to assess changes in genes expression. *MHC II*α and *MHC II*β mRNA levels in mIgM^+^ B lymphocytes were also examined by qRT-PCR after incubation with *L. lactis*, as mentioned in RNA isolation and Illumina sequencing section.

To investigate the effect of recombinant type I interferon 3 (rIFN I-3) on gene expression, mIgM^+^ B lymphocytes of the Japanese flounder were sorted from the pooled peripheral blood of four fish using the immunomagnetic bead separation method as previously described. The sorted mIgM^+^ B lymphocytes were resuspended in serum-free L-15 medium and cultured in 24-well plates, prior to incubation with rIFN I-3 (0.1 μg/ml) for 12 h. A pET-28a-derived his-tag protein was used as a negative control. After incubation with rIFN I-3, total RNA was extracted from mIgM^+^ B lymphocytes, and the total mRNA levels of Toll-like receptor 2 (*TLR2*), Nod-like receptor c (*NLRC*), *Stat1*, and *MHC II*α in the mIgM^+^ B lymphocytes were examined using qRT-PCR. All qRT-PCR primers were designed according to the corresponding sequences and are listed in Table S1.

### Preparation of Recombinant Proteins

The domains of Japanese flounder IFN I-3 (GenBank no. LC222627.1) were analyzed using the SMART program. IFN I-3 coding sequences (excluding the signal peptide-encoding region) were amplified by RT-PCR with the following primers (F: 5′-CCGGAATTCATGCCGCCCTGCACACTG-3′, EcoR I; R: 5′-ACGCGTCGAC TGTGAAACAGCTGTGGTGGTCC-3′, Sal I.). The PCR product was purified and digested with specific restriction enzymes prior to ligation into the pET-28a vector. The resulting recombinant pET-28a-IFN I-3 plasmid was used to transform *E. coli* (DE3), which were cultured in LB medium to the mid-logarithmic phase and induced by adding isopropyl β-D-1-thiogalactopyranoside following transformation. His-tagged rIFN I-3 was purified using the His Trap^TM^ HP Ni-Agarose column (GE Healthcare China, China) according to the manufacturer's instructions. Purified rIFN I-3 was then subjected to stepwise dialysis and refolded as previously described (24). The refolded proteins were treated with Triton X-114 to remove endotoxin and assessed by sodium dodecyl sulfate-polyacrylamide gel electrophoresis (SDS-PAGE). The residual endotoxin within the recombinant protein preparation was detected using the ToxinSensor^TM^ Chromogenic LAL Endotoxin Assay Kit (GenScript, China). Protein concentration was determined using the Bradford method.

### IFN I-3 Stimulation Assay

Leukocytes isolated from three individual fish were treated as three independent samples. The isolated leukocytes were suspended in serum-free L-15 medium and then transferred to 24-well culture plates (500 μl/well) at 5 × 10^6^ cells per well. The leukocytes were next incubated with Japanese flounder rIFN I-3 (50 ng/well, at a final concentration of 0.1 μg/ml) for 12 h at 22°C. The pET-28a-derived his-tag protein was administered to the negative control wells. The phagocytosis assay was subsequently performed as described, but the incubation time was adjusted to 1 h. Prior to carrying out the phagocytosis assay, *E. tarda* were labeled with FITC, as previously described (10). After incubation with FITC-labeled *E. tarda*, non-ingested *E. tarda* were removed by placing the cell suspensions on top of a glucose cushion (3 ml PBS, pH 7.3, with 3% (w/v) BSA and 4.5% (w/v) D-glucose) and centrifugated at 100 *g* for 10 min at 4°C. The phagocytic activity of mIgM^+^ B lymphocytes was determined using a BD Accuri C6 flow cytometer.

### Flow Cytometric Analysis of Intracellular Reactive Oxygen Species

Leukocytes isolated from three individual fish were treated as three independent samples. The isolated leukocytes were suspended in serum-free L-15 medium, and transferred to 24-well plates (500 μl/well) at 5 × 10^6^ cells per well. The leukocytes were incubated with Japanese flounder rIFN I-3 (50 ng/well, at a final concentration of 0.1 μg/ml) for 12 h at 22°C. The pET-28a-derived his-tag protein was added to the negative control wells. Leukocytes were labeled with the anti-Japanese flounder IgM mAb and the secondary Alexa Fluor® 647 goat anti-mouse IgG antibody (Invitrogen/Molecular Probes, USA). Finally, intracellular reactive oxygen species (ROS) were detected using the ROS assay kit (Beyotime, China), according to the manufacturer's instructions with some modifications. Briefly, the cells were cultured in serum-free L-15 medium containing 0.1% 2′,7′-dichlorodihydrofluorescein diacetate (DCFH-DA) at 22°C for 30 min, prior to being washed with serum-free L-15 medium three times and measurement on the BD Accuri C6 flow cytometer. The fluorescence intensity of DCFH-DA was detected to assess intracellular mIgM^+^ B lymphocyte ROS levels.

### Fludarabine Inhibition Assay

Leukocytes isolated from three individual fish were treated as three independent samples. The isolated leukocytes were suspended in serum-free L-15 medium and transferred to 24-well plates (500 μl/well) at 5 × 10^6^ cells per well. The leukocytes in each well were incubated with 20 μM fludarabine (Selleck, USA) for 2 h, with dimethyl sulfoxide administered to the negative control samples. Next, the leukocytes were incubated with rIFN I-3 (50 ng/well, at a final concentration of 0.1 μg/ml) for 12 h at 22°C. The phagocytic rate and intracellular ROS activity in pelleted leukocytes were then determined by flow cytometry, gating on mIgM^+^ B lymphocytes.

### Statistical Analysis

Statistical analysis was performed using Statistical Product and Service Solution (SPSS) software (version 20.0; IBM Corp., USA), and statistical significance was analyzed using independent-samples *t*-tests. The results are presented as mean ± S.D., and differences were considered significant at *p* < 0.05.

## Results

### The mIgM^+^ B Lymphocyte Phagocytic Ability

**Figure 1:**
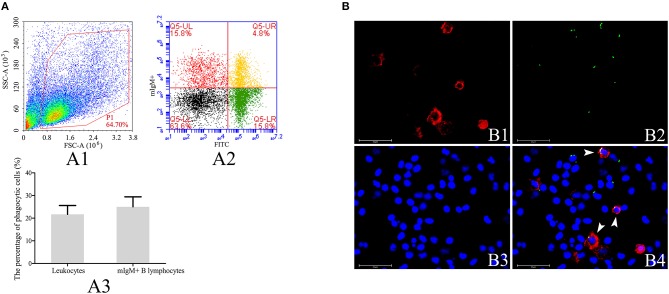
Analysis of phagocytosis of *L. lactis* by flounder mIgM^+^ B lymphocytes. **(A)** Flow cytometric analysis of phagocytosis of *L. lactis* by mIgM^+^ B lymphocytes of Japanese flounder. A1: Leukocytes ingested *L. lactis* in the peripheral blood were gated (P1) on a FSC/SSC. A2: Representative dot plots showing phagocytosis of *L. lactis* by mIgM^+^ B lymphocytes; Q5-LL: mIgM^−^ non-phagocytic leukocytes; Q5-LR: mIgM^−^ phagocytic leukocytes; Q5-UR: mIgM^+^ phagocytic B lymphocytes; Q5-UL: mIgM^+^ non-phagocytic B lymphocytes. A3: The phagocytosis of *L. lactis* by leukocytes and mIgM^+^ B lymphocytes were statistically analyzed using SPSS software. Error bars represent the standard deviation of three biological replicates. **(B)** The IIFA analysis of phagocytosis of *L. lactis* by mIgM^+^ B lymphocytes in peripheral blood. B1: Immunofluorescence-stained leukocytes with anti-IgM MAb and Alexa Fluor® 647 goat anti-mouse IgG. B2: FITC-labeled *L. lactis*. B3: Counter-stained cell nuclei in blue using DAPI. B4: The merged picture of B1, B2, and B3. The arrows indicated the mIgM^+^ B lymphocytes with ingested *L. lactis* (Bars = 25 μm).

To investigate the phagocytic ability of resident mIgM^+^ B lymphocytes, leukocytes isolated from the peripheral blood of the Japanese flounder were incubated with FITC-labeled *L. lactis*, prior to staining with a mAb specific for Japanese flounder IgM. The four quadrants of the flow cytometry plot, Q5-LL, Q5-LR, Q5-UL, and Q5-UR (Figure 1A), represent mIgM^−^ non-phagocytic leukocytes, mIgM^−^ phagocytic leukocytes, mIgM^+^ non-phagocytic B lymphocytes, and mIgM^+^ phagocytic B lymphocytes, respectively. Flow cytometric analysis showed that mIgM^+^ B lymphocytes phagocytosed *L. lactis*, and 25.0 ± 4.4% of the mIgM^+^ B lymphocytes had ingested *L. lactis* (Figure 1A). Consistent with the flow cytometric analysis results, phagocytosed *L. lactis* were also observed within a similar proportion of mIgM^+^ B lymphocytes by fluorescent microscopy (Figure 1B).

### Analysis of Sorted mIgM^+^ B Lymphocytes

**Figure 2:**
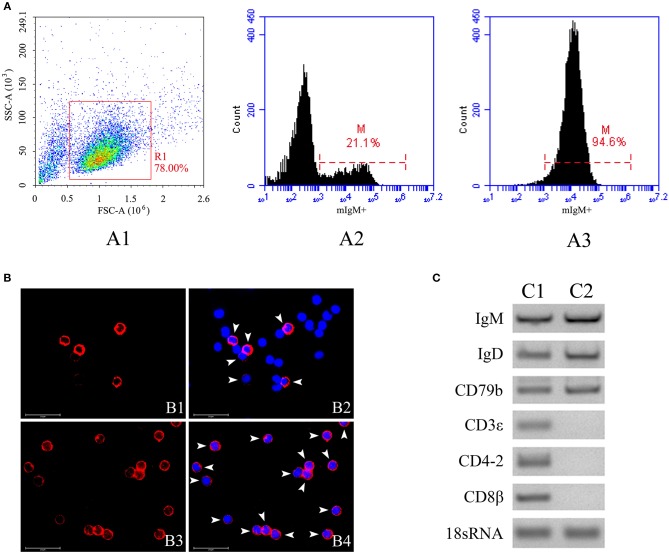
The purity analysis of mIgM^+^ B lymphocytes after sorting by flow cytometry, IIFA and RT-PCR. **(A)** Flow cytometric analysis of the purity of sorted mIgM^+^ B lymphocytes. The leukocytes in the peripheral blood were gated (R1) on FSC/SSC (A1), the fluorescence histogram showing the percentage of mIgM^+^ B lymphocytes (scale of M) in unsorted leukocytes (A2) and sorted mIgM^+^ B lymphocytes (A3). **(B)** IIFA analysis of the purity of sorted mIgM^+^ B lymphocytes. The unsorted leukocytes (B1 and B2) and sorted mIgM^+^ B lymphocytes (B3 and B4) were stained with MAb against IgM, cell nuclei were stained with DAPI; the red fluorescence (arrow) indicated the mIgM^+^ B lymphocytes (Bars = 25 μm). **(C)** RT-PCR analysis of expressions of marker genes for B cell (C1) and T cell (C2) in sorted mIgM^+^ B lymphocytes.

The mIgM^+^ B lymphocytes were magnetically sorted from peripheral blood, and the purity of the sorted fraction was determined by flow cytometry, IIFA, and RT-PCR. Flow cytometric analysis showed that the sorted mIgM^+^ B lymphocytes had a high (94.6%) degree of purity (Figure 2A). IIFA showed the presence of red fluorescent signals on the membranes of all sorted cells, also indicating high purity (Figure 2B). RT-PCR analysis showed that the expression of mIgM^+^ B cell (*IgM, IgD*, and *CD79b*) but not T cell (*CD3*ε, *CD4-2*, and *CD8*β) -associated marker genes were detected in sorted cells (Figure 2C), suggesting that the sorted mIgM^+^ B lymphocytes contained few cellular contaminants. Moreover, trypan blue exclusion assay results confirmed that the sorted mIgM^+^ B lymphocytes were viable.

### DEG Identification and Enrichment Analysis

**Figure 3:**
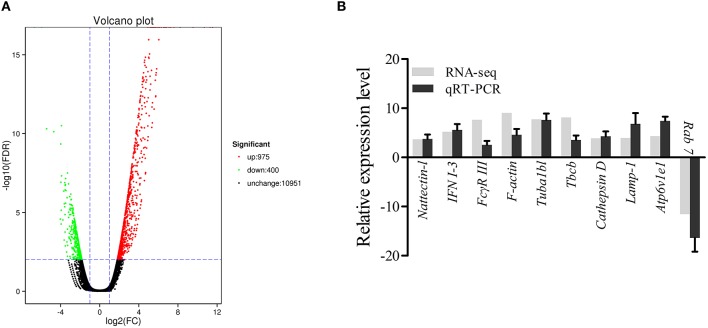
Analysis and validation of DEGs. **(A)** The “volcano plot” picture of DEGs between control group *L. lactis*-stimulated treatment group. Red spot, up-regulated; green spot, down-regulated; black spot, no difference in expression. **(B)** Validation of RNA-seq data by qRT-PCR analysis. X-axis, gene name; Y-axis, fold change in gene expression. The relative expression of 10 DEGs were determined by qRT-PCR and compared with the results of RNA-seq. Error bars represent the standard deviation of three technical replicates from a pool of sorted mIgM^+^ cells.

**Figure 4:**
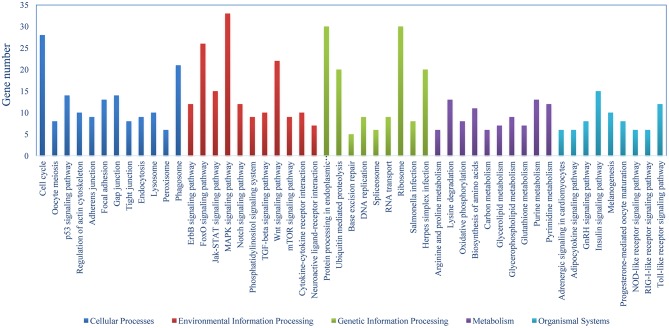
Pathway classification of DEGs in mIgM^+^ B lymphocytes. KEGG was used to assign all DEGs.

The DEGs were identified on the basis of an expression fold change ≥2 and an FDR < 0.01. A total of 1375 genes were differentially expressed in mIgM^+^ B lymphocytes following *L. lactis* stimulation *in vitro*; 975 upregulated and 400 downregulated genes were identified (Figure 3A). To analyze the cellular response of mIgM^+^ B lymphocytes to *L. lactis* stimulation, all DEGs were mapped to the KEGG database, and a total of 831 DEGs were successfully annotated. KEGG enrichment analysis showed that multiple pathways associated with phagocytosis (e.g., those involved in focal adhesion, the phagosome, and actin cytoskeleton regulation) were enriched (Figure 4). Moreover, many genes associated with phagolysosomal function were also upregulated after bacterial stimulation, such as *Cathepsin D*, lysosomal-associated membrane protein 1 (*Lamp-1*), and *Sec 61* (Table 1). Transcriptome analysis showed that the *MHC II*α and *MHC II*β genes were also upregulated, but their FDRs were >0.01 (Table 1). Therefore, qRT-PCR was further employed to analyze the expression profiles of *MHC II*α and *MHC II*β in mIgM^+^ B lymphocytes. The results revealed that *MHC II*α and *MHC II*β mRNA levels were increased following bacterial stimulation (Figure S1), which was consistent with the transcriptome data. The sequencing data derived from sorted mIgM^+^ B lymphocytes either in the presence or absence of *L. lactis* stimulation were deposited in the NCBI sequence read archive database under the accession numbers SAMN10104610 and SAMN10104609, respectively.

**Table 1:** Up-regulated genes involved in phagolysosome and antigen presentation.

**Gene name**	**Gene ID in transcriptome**	**log2FC**	**Regulated**	**FDR**
**PHAGOLYSOSOME**				
*Cathepsin D*	gene24035	1.96	Up	0.00526
*Lamp-1*	gene9086	1.98	Up	0.00436
*Atp6v1f*	gene22336	2.24	Up	0.00124
*Atp6v1e1*	gene520	2.11	Up	0.00190
*Atp2b1*	gene12803	2.60	Up	1.41E-05
*Sec 61*	gene7449	2.34	Up	0.000277
*M6pr*	gene17502	2.53	Up	4.58E-05
*Mpi*	gene2890	1.94	Up	0.00338
**ANTIGEN PRESENTATION**				
*MHC IIα*	gene19714	0.88	Up	0.967
*MHC IIβ*	gene19713	1.83	Up	0.0912

### DEG Validation by qRT-PCR

To validate the DEG data, 10 genes (9 upregulated and 1 downregulated), were randomly selected for qRT-PCR analysis. As shown in Figure 3B, the expression profiles for the 10 genes roughly coincided with the RNA-seq results, which confirmed the reliability of the transcriptome data.

### Recombinant IFN I-3 Expression and Purification

**Figure 5:**
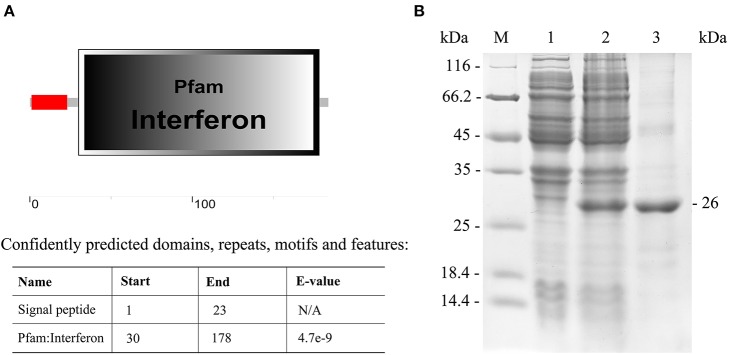
Analysis of IFN I-3 domain of Japanese flounder and recombinant expression. **(A)** Analysis of IFN I-3 domain of Japanese flounder using SMART program. The red line and big box represent signal peptide and PFAM domain, respectively. **(B)** SDS-PAGE analysis of expression and purification of rIFN I-3 protein of Japanese flounder using pET-28a vector by *E. coli* (DE3). Lane M, molecular mass marker; Lane 1, transformated *E. coli* without IPTG induction; Lane 2, transformated *E. coli* induced with IPTG; Lane 3, purified rIFN I-3 protein.

The IFN I-3 domains were analyzed using the SMART program, showing that the polypeptide was composed of a signal peptide (residues 1-23) and an interferon region (residues 30-178) (Figure 5A). The Japanese flounder IFN I-3 (excluding the region coding for its signal peptide) was expressed in *E. coli* (DE3) using a pET-28a plasmid system. SDS-PAGE analysis showed a distinct ~26-kDa band after isopropyl β-D-1-thiogalactopyranoside induction, which was consistent with the predicted molecular mass for the combination of the Japanese flounder IFN I-3 plus the pET-28a vector his-tag protein, confirming successful IFN I-3 expression (Figure 5B). After performing the Ni2^+^ affinity chromatography purification and stepwise dialysis refolding methods, high-purity rIFN I-3 protein was obtained (Figure 5B). Following Triton X-114 treatment, the endotoxin content was <0.05 endotoxin units per mg of purified rIFN I-3.

### IFN I-3 Enhances Phagocytosis by mIgM^+^ B Lymphocytes

**Figure 6:**
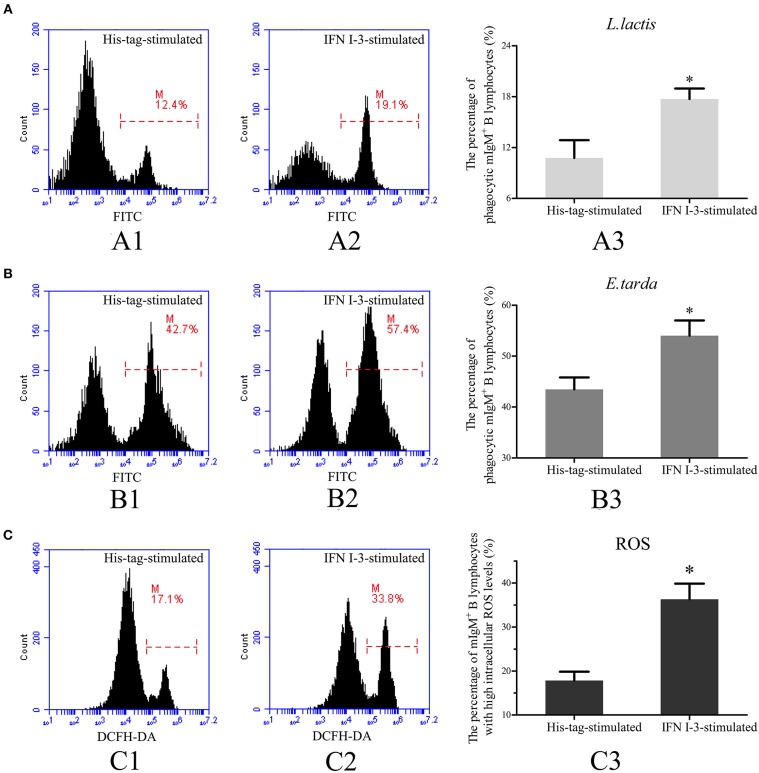
Flow cytometric analysis of phagocytosis and intracellular ROS activity by mIgM^+^ B lymphocytes of Japanese flounder with rIFN I-3 stimulation. **(A,B)** Phagocytosis of *L. lactis* **(A)** and *E. tarda* **(B)** by mIgM^+^ B lymphocytes with rIFN I-3 stimulation. The FITC fluorescence histogram showing the percentages of phagocytic mIgM^+^ B lymphocytes (scale of M) in His-tag-stimulated group (A1 and B1) and IFN I-3-stimulated group (A2 and B2). The data of phagocytosis of *L. lactis* (A3) and *E. tarda* (B3) by mIgM^+^ B lymphocytes with rIFN I-3 stimulation was analyzed using SPSS software, respectively. **(C)** Flow cytometric analysis of intracellular ROS levels in mIgM^+^ B lymphocytes with rIFN I-3 stimulation. The FITC fluorescence histogram showing the percentage of mIgM^+^ B lymphocytes with high intracellular ROS levels (scale of M) in His-tag-stimulated group (C1) and IFN I-3-stimulated group (C2). The data of intracellular ROS levels of mIgM^+^ B lymphocytes with rIFN I-3 stimulation (C3) were analyzed using SPSS software. The asterisk on the bars represents statistical significance of phagocytic rates and intracellular ROS levels compared with His-tag-stimulated groups (*p* < 0.05). All the error bars represent the standard deviation of three biological replicates.

Transcriptome analysis showed that IFN I-3 expression in mIgM^+^ B lymphocytes was significantly upregulated upon *L. lactis* stimulation, prompting further investigation into the role of IFN I-3 in mIgM^+^ B lymphocyte-mediated phagocytosis. Flow cytometric analysis revealed that on rIFN I-3 incubation, mIgM^+^ B lymphocytes acquired significantly higher *L. lactis* phagocytic activity (corresponding to a 164.4% increase), compared with the negative control group (Figure 6A). The phagocytosis of *E. tarda* by mIgM^+^ B lymphocytes was significantly higher than that of *L. lactis*. Furthermore, the phagocytic rate of *E. tarda* increased by 123.6% following rIFN I-3 stimulation (Figure 6B).

### IFN I-3 Enhances Intracellular ROS Production by mIgM^+^ B Lymphocytes

Following rIFN I-3 administration, intracellular ROS levels of mIgM^+^ B lymphocytes were determined by flow cytometry, measuring DCFH-DA fluorescence intensity. Flow cytometric analysis showed that the mIgM^+^ B lymphocytes of the Japanese flounder could be subdivided into distinct populations according to their intracellular ROS levels. In the negative control group, stimulated with the his-tag protein alone, 17.8 ± 2.0% of mIgM^+^ B lymphocytes had high intracellular ROS levels. In comparison, rIFN I-3 treatment significantly enhanced ROS production, with high levels observed in 36.2 ± 3.6% of the mIgM^+^ B lymphocytes (Figure 6C).

### Expression Profiles of Genes Related to Immune Function on IFN I-3 Stimulation

**Figure 7:**
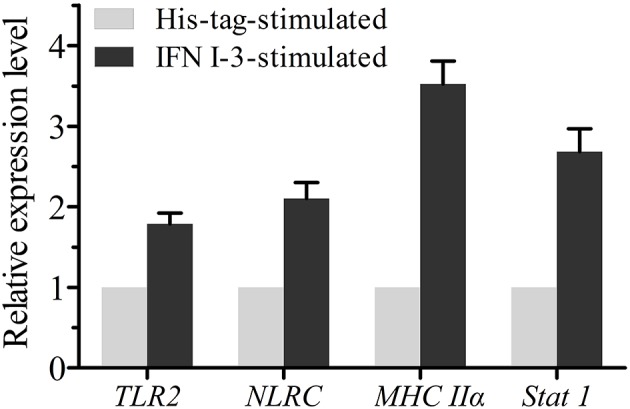
The qRT-PCR analysis of the expressions of several immune-related genes in mIgM^+^ B lymphocytes with rIFN I-3 stimulation. The mRNA levels of each immune-related gene were normalized to those of *18s rRNA*. For each gene, the mRNA level of the control groups was set as 1. Error bars represent the standard deviation of three technical replicates from a pool of sorted mIgM^+^ cells.

Expression changes of *TLR2, NLRC, Stat1*, and *MHC II*α mRNA in mIgM^+^ B lymphocytes were analyzed by qRT-PCR, following rIFN I-3 stimulation (Figure 7). The two pattern recognition receptor (PRR) genes *TLR2* and *NLRC*, as well as *Stat1* and *MHC Ii*α, were upregulated in mIgM^+^ B lymphocytes following incubation with rIFN I-3.

### Role of Stat1 in mIgM^+^ B Lymphocyte-Mediated Phagocytosis

**Figure 8:**
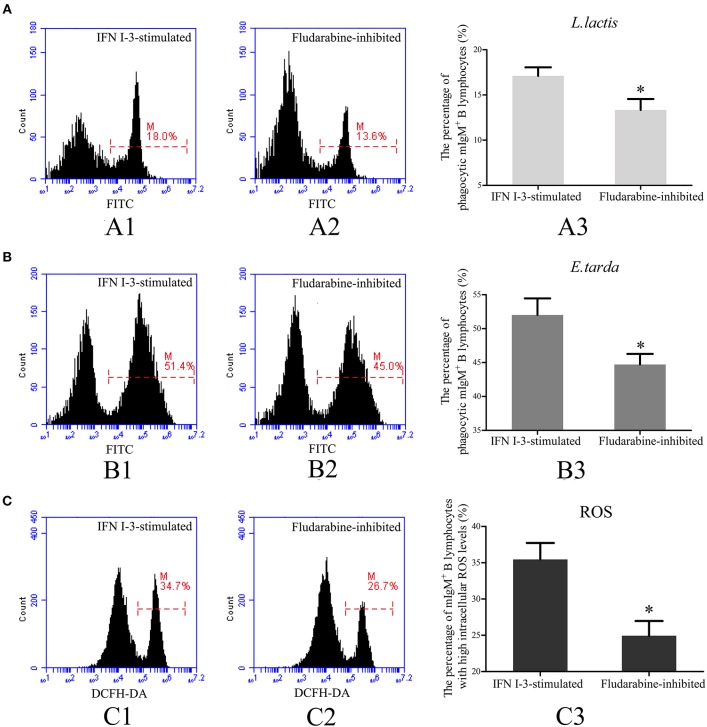
Flow cytometric analysis of phagocytosis and intracellular ROS activity of rIFN I-3-stimulated mIgM^+^ B lymphocytes with fludarabine treatment. **(A,B)** Phagocytosis of *L. lactis* **(A)** and *E. tarda* **(B)** by rIFN I-3-stimulated mIgM^+^ B lymphocytes with fludarabine treatment. The FITC fluorescence histogram showing the percentages of phagocytic mIgM^+^ B lymphocytes (scale of M) in IFN I-3-stimulated group (A1 and B1) and fludarabine-inhibited group (A2 and B2). The data of phagocytosis of *L. lactis* (A3) and *E. tarda* (B3) by mIgM^+^ B lymphocytes with fludarabine treatment was analyzed using SPSS software, respectively. **(C)** Flow cytometric analysis of intracellular ROS levels in rIFN I-3-stimulated mIgM^+^ B lymphocytes with fludarabine treatment. The FITC fluorescence histogram showing the percentage of mIgM^+^ B lymphocytes with high intracellular ROS levels (scale of M) in IFN I-3-stimulated group (C1) and fludarabine-inhibited group (C2). The data of intracellular ROS levels of mIgM^+^ B lymphocytes with fludarabine treatment (C3) were analyzed using SPSS software. The asterisk on the bars represents statistical significance of phagocytic rates and intracellular ROS levels compared with IFN I-3-stimulated groups (*p* < 0.05). All the error bars represent the standard deviation of three biological replicates.

To investigate the role of Stat1 in IFN I-3-stimulated regulation of mIgM^+^ B lymphocyte phagocytosis, fludarabine was employed to inhibit Stat1 phosphorylation. Following treatment with rIFN I-3 alone, 17.1 ± 1.0% of mIgM^+^ B lymphocytes were able to phagocytose *L. lactis*. In the presence of rIFN I-3 and fludarabine, however, significantly lower phagocytosis activity of 13.3 ± 1.2% was observed (Figure 8A). Similarly, phagocytic rate of *E. tarda* by mIgM^+^ B lymphocytes decreased from 52.1 ± 1.8 to 44.7 ± 1.6% after administering fludarabine to rIFN I-3-stimulated mIgM^+^ B lymphocytes (Figure 8B). Flow cytometric analysis revealed that the intracellular ROS levels of rIFN I-3-stimulated mIgM^+^ B lymphocytes were significantly decreased following fludarabine treatment; the percentage of mIgM^+^ B lymphocytes with high intracellular ROS levels dropped from 36.2 ± 3.6 to 24.9 ± 2.0% (Figure 8C).

## Discussion

B cells are key players in the immune response, responsible for effective antigen presentation and antibody secretion, but they were previously thought to lack phagocytic activity (5, 25). Over the past decade, accumulating evidence suggests that B cells can also carry out phagocytosis, at least in certain species of teleosts, amphibians, reptiles, and mammals (6, 11, 12, 14). The present study confirms that mIgM^+^ B lymphocytes of the Japanese flounder actively ingest and phagocytose *L. lactis* and *E. tarda*. To further elucidate the phagocytosis mechanism, Japanese flounder-derived mIgM^+^ B lymphocytes were sorted from peripheral blood leukocytes using the immunomagnetic bead separation method. A high level of purity (94.6%) for the sorted mIgM^+^ B lymphocytes was confirmed by flow cytometry, IIFA, and RT-PCR techniques. Trypan blue exclusion assays confirmed that the sorted mIgM^+^ B lymphocytes were viable. These results collectively demonstrate that the sorted mIgM^+^ B lymphocytes were of sufficient purity and viability for transcriptomics analysis. Previous studies reported that IgM can bind to the Fc-receptor on the surface of monocytes and macrophages (26, 27), which may contaminate sorted mIgM^+^ B cells. Therefore, we cannot discount the possibility that other cell types (accounting for the remaining 5.4% of the sorted cells) or contamination with IgM^+^ monocytes and macrophages may have influenced the RNA-seq results.

To obtain deeper insight into the mechanisms of B cell-mediated phagocytosis, transcriptomics analysis was initially used to investigate the response of mIgM^+^ B lymphocytes upon *L. lactis* stimulation *in vitro*. KEGG analysis showed that many mIgM^+^ B lymphocyte-associated DEGs were enriched in focal adhesion, actin cytoskeleton regulation, and phagosome pathways. These pathways are all involved in phagocytosis, suggesting that the phagocytic activity of mIgM^+^ B lymphocytes was activated upon *L. lactis* stimulation. Moreover, transcriptome analysis revealed that many genes involved in phagolysosome function and antigen presentation were upregulated upon *L. lactis* stimulation, highlighting the ability of mIgM^+^ B lymphocytes to degrade internalized bacteria and present processed antigenic peptides, much like macrophages. Mouse B1 cells can destroy internalized bacteria and present processed antigenic peptides to CD4^+^ T cells after phagocytosis (12). Similarly, zebra fish (*Danio rerio*) B cells exhibit a potent ability to present both soluble antigen and bacterial particles to prime naive CD4^+^ T cells (15).

Cytokines are a class of low molecular weight, highly active glycoproteins that play a vital role in mediating and regulating the inflammatory response. In mammals, the IFN I cytokine is a major component of innate immunity. The IFN I-mediated response is traditionally associated with viral infections, but recent studies indicate that it may also be effective in eliminating bacteria (28, 29). A previous study showed that IFN-α enhances monocyte Fc-dependent phagocytosis in part by increasing the number of Fc receptors (30). Another group reported that IFN-α also affected the phagocytosis of non-opsonized *E. coli* by mouse peritoneal macrophages *in vitro* (31). Our transcriptome analysis also demonstrated that *IFN I-3* was upregulated in the mIgM^+^ B lymphocytes of the Japanese flounder upon *L. lactis* stimulation *in vitro*. Further analysis found that rIFN I-3 increased the ability of mIgM^+^ B lymphocytes to phagocytose both *L. lactis* and *E. tarda*. Moreover, the intracellular ROS levels of mIgM^+^ B lymphocytes were enhanced by stimulation with rIFN I-3. Bacterial killing through intracellular ROS generation is a highly efficient non-specific cellular defense mechanism (32). Previous studies have shown that intracellular ROS also contribute to the bactericidal activity of phagocytes, which is critical for preventing bacterial infection (33, 34). Collectively, this evidence demonstrates that IFN I-3 enhances both the phagocytic and intracellular bactericidal abilities of mIgM^+^ B lymphocytes.

The innate immune system employs PRRs to recognize microbial pathogens by binding to pathogen-associated molecular patterns (PAMPs) (35). TLR2 and NLRC are two important PRRs that play crucial roles in the innate immune response, particularly in enhancing bacterial clearance via phagocytosis (36–38). In this study, qRT-PCR analysis showed that *TLR2* and *NLRC* were upregulated in the mIgM^+^ B lymphocytes of the Japanese flounder on IFN I-3 stimulation, implying that IFN I-3 may increase the expressions of certain PRRs to promote B-cell mediated phagocytosis. In addition, *MHC II*α was upregulated after IFN I-3 stimulation, which suggests that IFN I-3 also enhance antigen presentation by mIgM^+^ B lymphocytes.

The Jak/Stat pathway is a major signaling pathway activated by IFN I that mediates the immune response by upregulating the expression of IFN-stimulated genes (39, 40). Previous studies have shown that IFN I signals are transmitted through membrane-associated Tyk2 or Jak1 kinases, which then signal downstream through Stat1/Stat1 or Stat1/Stat2 complexes (41, 42). In the present study, *Stat1* was upregulated upon IFN I-3 stimulation of Japanese flounder-derived mIgM^+^ B lymphocytes. Flow cytometric analysis showed that the enhancing effect of IFN I-3 on the phagocytic and intracellular ROS activity of mIgM^+^ B lymphocytes was suppressed by fludarabine. As an inhibitor of Stat1 phosphorylation, fludarabine is routinely used to study the function of Stat1 in many settings (43–45). We speculated that as demonstrated in mammals, Stat1 may regulate IFN I-3-stimulated phagocytosis in mIgM^+^ B cells from the Japanese flounder. Regrettably, we were not able to directly determine the phosphorylation status of Stat1 following fludarabine treatment due to the unavailability of a specific antibody against the phosphorylated form of Japanese flounder-specific Stat1. Besides inhibiting Stat1 phosphorylation, fludarabine can also inhibit RNA transcription and deplete cellular ATP levels, factors that may influence the phagocytic activity of treated cells (46). Further studies are needed to clarify the role of Stat1 on the regulatory effect of IFN I-3 during mIgM^+^ B lymphocyte-mediated phagocytosis.

In summary, we evaluated the phagocytic capacity of mIgM^+^ B lymphocytes from the Japanese flounder. Transcriptome profiling of the *in vitro* anti-*L. lactis* response mediated by Japanese flounder IgM^+^ B lymphocytes revealed enrichment of multiple pathways associated with phagocytosis, and expression levels of many phagolysosome-associated genes were upregulated. Our findings suggest that mIgM^+^ B lymphocytes may possess the ability to degrade ingested bacteria and present processed antigenic peptides to other immune cells. Finally, IFN I-3 enhances both the phagocytic and intracellular bactericidal abilities of mIgM^+^ B lymphocytes, with Stat1 potentially playing an important role. We envisage that these findings will broaden our understanding of the regulatory mechanisms underlying B cell-mediated phagocytosis.

## Ethics Statement

This study was carried out in strict accordance with the ethical standards and the guidelines of Regulations for the Administration of Affairs Concerning Experimental Animals documented by the State Science and Technology Commission of Shandong Province. This study was also approved by the Committee of the Ethics on Animal Care and Experiments at Ocean University of China. Fish were anesthetized with ethyl 3-amino-benzoate-methanesulfonic acid (MS222) before sacrificing and handling.

## Author Contributions

XT and SY contributed to the conception and design of this study, performed most of experiments and statistical analysis, drafted, and revised the manuscript. JX and XS participated in the design of the study, helped, and analyzed the experiments and data. WZ designed the study, edited the manuscript, and provided reagents and experiment space. All the authors read and approved this version of the final manuscript and confirm the integrity of this work.

### Conflict of Interest Statement

The authors declare that the research was conducted in the absence of any commercial or financial relationships that could be construed as a potential conflict of interest.
