# Supplementary material for: Transcriptome Analysis of Immune Response of mIgM+ B Lymphocytes in Japanese Flounder (Paralichthys olivaceus) to Lactococcus lactis in vitro Revealed That IFN I-3 Could Enhance Their Phagocytosis
Source: Front Immunol. 2019 Jul 16;10:1622. doi: 10.3389/fimmu.2019.01622 (PMC6646603; doi:10.3389/fimmu.2019.01622)
Supplement: Supplementary file 1 [file Data_Sheet_1.doc]

***Supplementary Material***

# Supplementary Figures and Tables

## Supplementary Figures

**
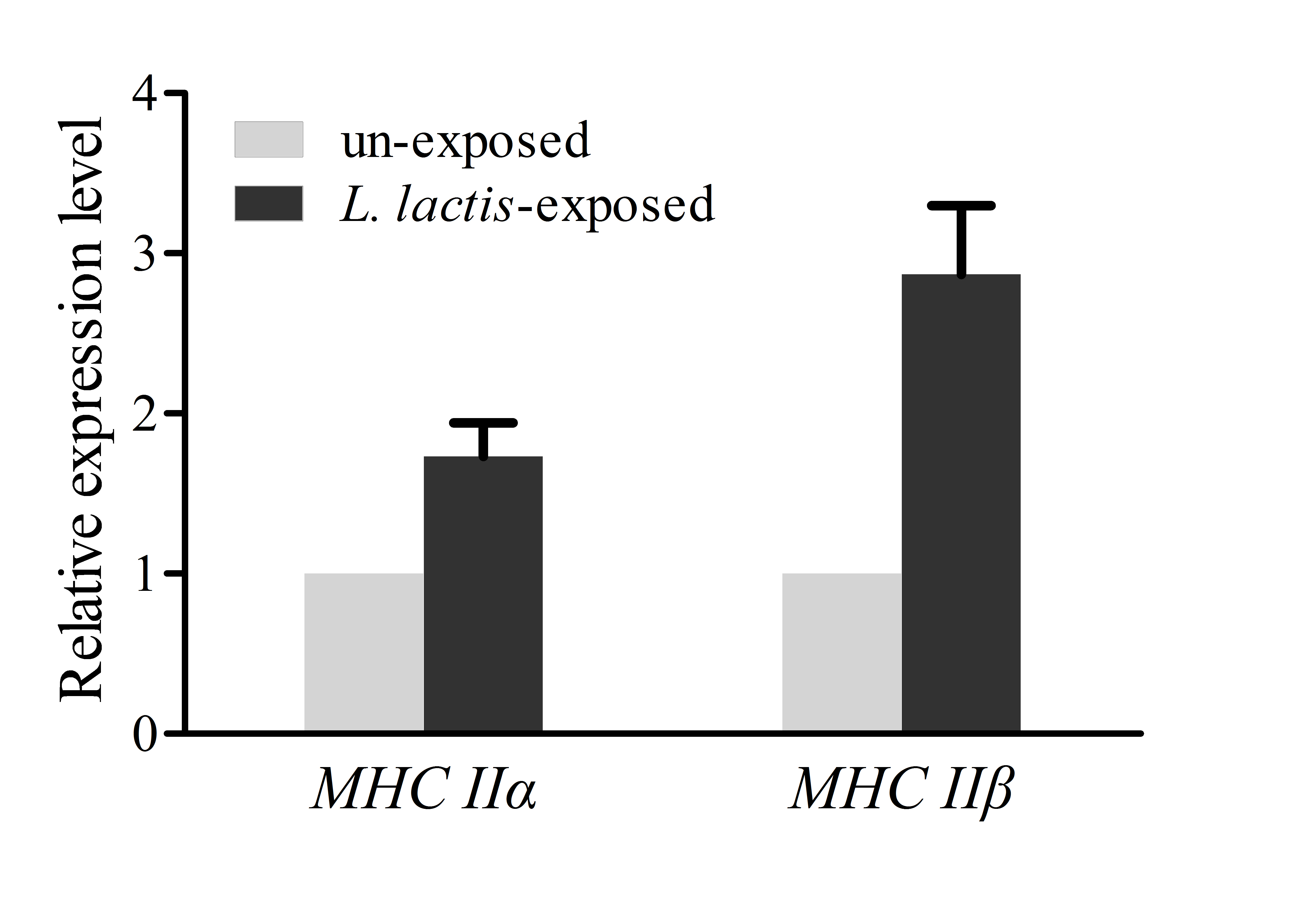
**

**Figure S1. The qRT-PCR analysis of the expression of MHC II in mIgM^+^ B lymphocytes with *L.lactis* stimulation.** The mRNA levels of each gene were normalized to those of 18S rRNA. For each gene, the mRNA level of the control group was set as 1. Results are expressed as means ± SD (n=3). The data were representative of eight individual fish from one experiments with technical replicates.

## Supplementary Table

**Table S1 Detailed information of primers used for qRT-PCR and RT-PCR.**

| **Gene name** | **Forward primer** | **Reverse primer** |
| --- | --- | --- |
| *Nattectin-l* | CCGAAGATGTGGGCTGAA | CTGAGGGAAGTGGTGGGT |
| *IFN I-3* | AGGTATTATGGGTGGTGTTTG | CATCGGTCAGCAGTCGGT |
| *FcR r III* | CTGGGACGCAGACAGACTTA | TATTGGAGGTGGCAGATGAG |
| *F-actin* | ACTGCTACAAACCCAAAC | CCTTCATAGAGGCAAATAA |
| *Tuba1bl* | ATGCGTGAGTGTATCTCCGTGC | TCTCCTCCTCCGATGGTCTT |
| *Tbcb* | GCATTACCAGCACCATCTAC | AAGCGTCATTGTCATTCATT |
| *Cathepsin D* | TGTGGATTCTGGGAGATGTA | TTGCTAAAGACGGTTTGATT |
| *Lamp-1* | ATAAAGGGAATGTTTCTGGC | CGTGCGTTTCATCACTAATC |
| *Atp6v1e1* | GCTCCTCCTCCTCTGTGGT | CTCCTCGGTTTGGTCCTTT |
| *Rab 7* | CACTGAACATAGACGGACAC | GAGAAACTGGCTTTGGAC |
| *TLR2* | CTGCGGTGTAGCGTTAGTGG | CGAAGGCATCATAGGAAAGC |
| *NLRC* | GGTCATTTCATCGCTTATCA | AAAGAAAGACAGCGTGCC |
| *Stat1* | CCATCATGGGTTTCATCTGT | TTGTCGTGTACGTCGTGTTC |
| *MHC IIα* | ACAGGGACGGAACTTATCAACG | TCATCGGACTGGAGGGAGG |
| *MHC IIβ* | CTCCCTCTTCTTCATCACGG | TCCAACGAACTTCCCCACA |
| *IgM* | GTCCACAAATTACCCTCCAA | AGCCGATTCAGGCAAGTC |
| *IgD* | TTTCAAGCCAACGATAAG | AAGTTCCACAGAAGGGTC |
| *CD79b* | GAGGTAAAGGGAGCAAGG | CATCAGAATAGCGACAGC |
| *CD3ε* | ATGACGCTCCTCCTGTAT | TTCGCATCACTATTATCCTT |
| *CD4-2* | TTAGAAACCCTCCCTGTCCACG | AGGGCAGTTATGATGAAACGAATG |
| *CD8β* | GCAATACTCCCAATCCCA | AGTCCCAGGCTTCCATAC |
| *18s rRNA* | GGTCTGTGATGCCCTTAGATGTC | AGTGGGGTTCAGCGGGTTAC |
